# The effect of enteral nutrition on adipokines in patients with acute pancreatitis

**DOI:** 10.1017/jns.2015.20

**Published:** 2015-10-12

**Authors:** Sarah J. L. McKenzie, Rakesh Premkumar, Kathryn J. Askelund, Sayali A. Pendharkar, Anthony R. J. Phillips, John A. Windsor, Maxim S. Petrov

**Affiliations:** Department of Surgery, University of Auckland, Auckland, New Zealand

**Keywords:** Enteral nutrition, Adipokines, Acute pancreatitis, Adipose tissue, Human and clinical nutrition, AP, acute pancreatitis, APACHE, Acute Physiology and Chronic Health Evaluation, EN, enteral nutrition, MD, mean difference, NBM, nil-by-mouth, T2DM, type 2 diabetes mellitus

## Abstract

The mechanism behind the beneficial effects of enteral nutrition (EN) for patients with acute pancreatitis (AP) is largely unknown. Adipokines, as mediators of metabolism and inflammation, may be a possible mechanism. The study aimed to investigate the effect of EN on adipokines early in the course of AP. Patients with AP were randomised to EN or nil-by-mouth (NBM). Blood samples were taken on the first 4 d of admission and adipokine concentrations for adiponectin, leptin, omentin, resistin and visfatin were determined by ELISA assays. A linear mixed model analysis was run to determine differences in adipokine concentrations between the two study groups. A total of thirty-two patients were included in the study. Omentin concentrations were significantly higher in patients who received EN compared with NBM across the first 4 d of admission (mean difference: 11·6 (95 % CI 1·0, 22·3) ng/ml; *P* = 0·033). Leptin concentrations were significantly higher in patients who received EN compared with NBM after adjusting for age, sex and BMI (mean difference: 2·3 (95 % CI 0·1, 4·5) ng/ml; *P* = 0·037). No significant difference in adiponectin, resistin or visfatin concentrations were observed between the two study groups. EN significantly increases omentin and leptin concentrations in AP. Future research should be directed towards understanding whether these adipokines are responsible for the therapeutic benefits of EN.

Acute pancreatitis (AP) is one of the most common causes for gastrointestinal-related hospitalisations, with more than 270 000 admissions in the USA alone per year^(^^1^^)^. With an increasing incidence of AP worldwide, and estimated costs of US$2·6 billion per annum, AP places a significant burden on health care systems^(^^1^^,^^2^^)^.

Enteral nutrition (EN) is a well-established treatment modality for patients with AP^(^^3^^–^^5^^)^. It provides many clinical benefits such as reduced risk of infectious complications and multi-organ dysfunction syndrome, reduced length of hospital stay, and mortality^(^^6^^–^^13^^)^. The common belief is that EN is beneficial because it prevents gut barrier dysfunction, maintains mucosal integrity and reduces bacterial translocation^(^^14^^–^^17^^)^. However, a recent comprehensive systematic review of studies that used standard EN in patients with AP found that significant improvements in gut barrier function had been observed in only half of the randomised controlled trials^(^^18^^)^. Hence, it is conceivable that EN exerts its effect via a mechanism other than improvement in gut barrier function, at least in some patients. A possible alternative mechanism for the therapeutic benefit of EN is through adipokines – adipose tissue-derived cytokines^(^^19^^,^^20^^)^. A recent clinical study of sixteen patients with Crohn's disease found that patients who received exclusive EN for 4 weeks prior to ileal resection had lower leptin concentrations and higher adiponectin concentrations compared with those who received no specific nutrition therapy^(^^21^^)^. EN was also observed to restore adipocyte morphology and reduce inflammation in mesenteric fat associated with intestinal injury in Crohn's disease^(^^21^^)^. The relationship between adipokines and EN has not been studied in patients with AP before. Most studies in AP patients have evaluated adipokines as severity markers and have been usually limited to just one to three adipokines^(^^20^^,^^22^^–^^26^^)^.

The aim of the present study was to investigate the effect of EN, as compared with nil-by-mouth (NBM), on levels of adiponectin, leptin, omentin, resistin and visfatin, from before randomisation to 72 h after randomisation in patients with AP.

## Methods

### Study design

This was a substudy of the randomised controlled MIMOSA (MIld to MOderate Acute Pancreatitis: Early naSogastric Tube Feeding Compared With pAncreas Rest) trial, conducted at Auckland City Hospital, Auckland, New Zealand^(^^27^^)^. The clinical outcomes have been reported elsewhere^(^^27^^)^. The New Zealand Northern X Human Ethics Committee approved the study protocol (NTX/08/11/107). The study protocol was also registered at www.clinicaltrials.gov (NCT01128478).

### Inclusion criteria

Patients were included in the study if they had a confirmed diagnosis of AP, were at least 18 years of age or older, and provided written informed consent.

### Exclusion criteria

Patients were excluded from the study if they had/were:

>96 h after onset of symptoms;
≥24 h after hospital admission;
Severe or critical AP;
Chronic pancreatitis;
Post-endoscopic retrograde cholangiopancreatography pancreatitis;
Intra-operative diagnosis of AP;
Pregnant;
Malignancy;
Received nutrition before randomisation;
Previously enrolled into the trial.

### Study groups

There were two study groups: (1) intervention patients, who received EN by a nasogastric tube within 24 h of admission; (2) control patients (NBM). Patients in both study groups were transitioned to stepwise oral refeeding when the treating teams deemed it appropriate.

### Endpoint

Endpoints were differences between the two study groups in any of the following five adipokine concentrations: adiponectin, leptin, omentin, resistin and visfatin, across or between the following four time points: before randomisation (within 24 h of admission), 24, 48 and 72 h after randomisation.

### Plasma collection and storage

Venous blood was collected into citrate phosphate dextrose-adenine tubes. After separation the plasma was aliquoted into low binding Axygen microtubes and then snap-frozen in liquid N_2_ before being stored in a −80°C freezer until use. Data were obtained for the first four consecutive days of hospital admission: baseline (within 24 h of admission), and at 24, 48 and 72 h after randomisation.

### Laboratory techniques – ELISA

Leptin and omentin concentrations were measured using Millipore ELISA kits according to the manufacturer's instructions. For leptin, the Assay Procedure II Human Leptin (sensitive) assay was used. Visfatin concentrations were measured using an MBL ELISA kit according to the manufacturer's instructions.

Briefly, these were sandwich ELISA assays based on capture of leptin by polyclonal rabbit anti-human leptin antibody and of omentin by anti-omentin IgG, bound to the wells of a microtitre plate coated with anchor antibodies. This was followed by simultaneous binding of a second biotinylated antibody to the leptin or omentin. For visfatin, the plate was coated with a monoclonal antibody to human visfatin and the secondary antibody was an anti-human rabbit polyclonal. The plates were washed and horseradish peroxidase (HRP) was conjugated to the immobilised biotinylated antibodies. Free enzyme was washed away and the antibody–enzyme conjugates were quantified by monitoring HRP activity in the presence of tetramethylbenzidine. The enzyme activity was measured spectrophotometrically by the increased absorbency at 450 nm in a plate reader (Enspire, 2300 Multilabel Reader; Perkin Elmer), corrected from the absorbency at 590 nm, after acidification of formed products.

Adiponectin and resistin were quantified simultaneously using the Multiplex Millipore ELISA kit according to the manufacturer's instructions. This assay is based on fluorescent dye colour-coded microspheres coated with specific capture antibodies. Analytes from test samples were captured by the beads, after which a biotinylated detection antibody was added. The reaction mixture was incubated with streptavidin–phycoerythrin conjugate, the reporter molecule, to complete the reaction on the surface of each microsphere. The plate was run on a Luminex 100^™^ IS microplate reader (Luminex Corporation). Median fluorescent intensity data were analysed using a five-parameter logistic equation to quantify the concentrations of adiponectin and resistin.

### Data storage and statistical analysis

All data were stored in a secure encrypted central Internet-based database MACRO (InferMed Limited). The statistical analysis was done using SPSS for Windows version 21 (SPSS Inc.).

Comparison of three or more sets of repeated measures was performed using the linear mixed model. The diagonal covariance type model was used when conducting the analysis for each of the five adipokines during hospital admission. Group and time were fit as the fixed variables while adipokine concentration was fitted as the dependent variable. A main effects type III sum of squares model was built to obtain the most robust and conservative output. The fit of the model was determined by the log 2 value and the best fit model selected accordingly.

A univariate linear mixed model analysis was then run to assess whether there was a difference in adipokine concentrations between the two study groups during the study period. Pairwise comparison was used to determine the mean difference (MD) between the two study groups across the four time points. To investigate whether potential confounders (age, sex, BMI, and Acute Physiology and Chronic Health Evaluation (APACHE) II score at admission) had an impact on adipokine concentrations, a main effects type III sum of squares model was built with time and sex fitted as factors, and age, BMI and APACHE II score at admission fitted as covariates. Confounders that showed a significant effect were then analysed simultaneously to compare the overall adjusted MD of adipokine concentrations between the EN and NBM groups.

Difference between the two study groups was expressed as MD with the corresponding 95 % CI. The *F* statistic was used to determine the equality of variances between the two study groups. A *P* value <0·05 was accepted as statistically significant.

## Results

### Baseline characteristics

A total of seventy-eight consecutive patients with AP were admitted to Auckland City Hospital during the study period. Of these, thirty-three patients met at least one of the exclusion criteria, ten declined to participate, and adipokine data were unavailable for three patients. The entire study cohort consisted of thirty-two patients, of which fifteen patients were randomised to the EN group and seventeen patients were randomised to the NBM group. At baseline, the two groups did not have any significant differences in terms of demographic, anthropometric and clinical data (Table 1).

**Table 1.** Baseline characteristics of patients in the enteral nutrition (EN) group and the nil-by-mouth (NBM) group (Number of patients and percentages; medians and interquartile ranges)

	EN group (*n* 15)		NBM group (*n* 17)		
	*n*	%	*n*	%	*P*
Age (years)					0·223
Median	42		53		
Interquartile range	36–60		38–71		
Sex					0·507
Male	8	53	7	41	
Female	7	47	10	59	
BMI (kg/m^2^)					0·774
Median	26		25		
Interquartile range	24–29		23–27		
Time from first symptoms to hospital admission (h)					0·568
Median	12		18		
Interquartile range	6–24		6–30		
Diabetes mellitus	4	27	2	12	0·296
Aetiology					0·627
Alcoholic	3	20	5	29	
Biliary	9	60	9	53	
Unknown	3	20	3	18	
ASA co-morbidity class					0·295
Class I	9	60	5	29	
Class II	2	13	8	47	
Class III	4	27	3	18	
Class IV	0	0	1	6	
HAPS score					0·491
1	3	20	5	29	
2	10	67	5	29	
3	2	13	7	42	
APACHE II score					0·339
Median	6		7		
Interquartile range	2–8		2–11		

ASA, American Society of Anaesthesiologists; HAPS, Harmless Acute Pancreatitis Score; APACHE II, Acute Physiology and Chronic Health Evaluation II.

### Univariable analysis

**Fig. 1.**
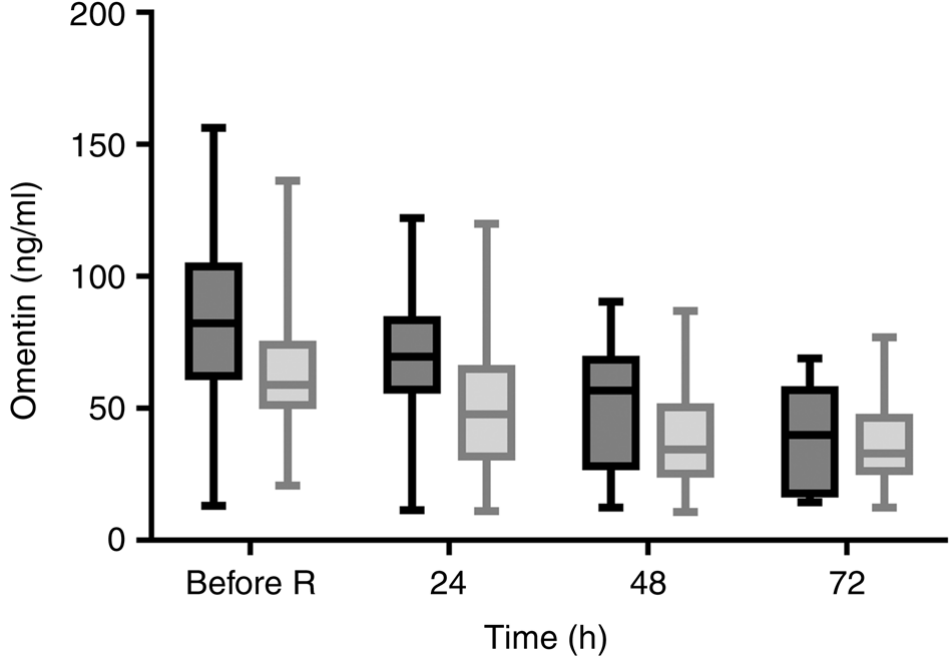
Omentin concentrations for the enteral nutrition group (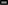; *n* 15) and the nil-by-mouth group (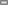; *n* 17) at each time point. Before R, before randomisation. Box and whisker plots represent medians and ranges. Time had a significant effect on omentin concentration across the study period (*P* < 0·001).

A significant difference in omentin concentrations was observed between the two study groups, whereby omentin concentrations were significantly higher in the EN group than the NBM group (MD: 11·6 (95 % CI 1·0, 22·3) ng/ml; *P* = 0·033) (Table 2). Time was observed to have a significant effect on omentin concentration (*P* < 0·001) across the study period, whereby omentin concentrations were elevated early during admission, then decreased with time in both study groups (Fig. 1). Omentin concentrations were significantly different between the two study groups between the following time points: from before randomisation to 48 h after randomisation (MD: 27·4 (95 % CI 7·3, 47·6) ng/ml; *P* = 0·002), before randomisation to 72 h after randomisation (MD: 35·7 (95 % CI 14·3, 57·0) ng/ml; *P* < 0·001), and from 24 to 72 h after randomisation (MD: 23·8 (95 % CI 3·1, 44·5) ng/ml; *P* = 0·015). No significant difference in omentin concentrations between the two study groups was observed from before randomisation to 24 h after randomisation (MD: 11·9 (95 % CI −7·9, 31·6) ng/ml; *P* = 0·651), from 24 to 48 h after randomisation (MD: 15·6 (95 % CI −3·9, 35·0) ng/ml; *P* = 0·201), and from 48 to 72 h after randomisation (MD: 8·2 (95 % CI −12·8, 29·3) ng/ml; *P* = 1·000).

**Table 2.** Univariable analysis of adipokine concentrations across the four time points in the study groups (Mean values and standard deviations; mean differences (MD) and 95 % confidence intervals)

	EN group		NBM group		Difference			
Adipokine	Mean	sd	Mean	sd	MD	95% CI	*F*	*P*
Adiponectin (μg/ml)	45·8	22·6	44·5	22·2	1·3	−10·9, 13·5	0·044	0·834
Leptin (ng/ml)	5·5	5·1	5·0	6·6	0·5	−2·1, 3·0	0·143	0·706
Omentin (ng/ml)	60·5	32·7	48·9	26·9	11·6	1·0, 22·3	4·691	0·033
Resistin (ng/ml)	13·0	10·0	14·1	11·3	−1·1	−5·3, 3·1	0·263	0·609
Visfatin (ng/ml)	3·4	1·2	3·1	1·8	0·2	−0·4, 0·8	0·620	0·433

EN, enteral nutrition; NBM, nil-by-mouth.

No significant differences were observed in adiponectin, leptin, resistin and visfatin concentrations between the two study groups (MD: 1·3 (95 % CI −10·9, 13·5) μg/ml; *P* = 0·834; MD: 0·5 (95 % CI −2·1, 3·0) ng/ml; *P* = 0·706; MD: −1·1 (95 % CI −5·3, 3·1) ng/ml; *P* = 0·609; and MD: 0·2 (95 % CI −0·4, 0·8) ng/ml; *P* = 0·433, respectively) across the four time points (Table 2). Time was not observed to have a significant effect on adiponectin, leptin, resistin and visfatin concentrations across the entire cohort (*P* = 0·866, *P* = 0·637, *P* = 0·949 and *P* = 0·388, respectively).

### Multivariable analysis

The following confounders were significantly associated with omentin concentrations: age (*P* = 0·031), BMI (*P* = 0·010), and APACHE II score at admission (*P* < 0·001). After adjusting for age, BMI and APACHE II score simultaneously, the MD in omentin concentrations between the two groups increased and remained significant, whereby omentin concentrations were higher in the EN group than in the NBM group (adjusted MD: 16·7 (95 % CI 6·5, 26·9) ng/ml; *P* = 0·002).

Three confounders were significantly associated with leptin concentrations: age (*P* = 0·045), sex (*P* = 0·006) and BMI (*P* < 0·001). After adjusting for age, sex and BMI simultaneously, leptin concentrations were observed to increase in significance in the EN group compared with the NBM group across all four time points (adjusted MD: 2·3 (95 % CI 0·1, 4·5) ng/ml; *P* = 0·037).

A significant inverse correlation was observed between BMI and adiponectin concentrations across the entire cohort (*P* = 0·029). No significant effect of age (*P* = 0·441), sex (*P* = 0·321) or APACHE II score at admission (*P* = 0·252) on adiponectin concentrations was observed in the entire cohort. Further analysis showed that BMI was not significantly associated with adiponectin concentrations between the two study groups (MD: 1·68 (95 % CI −9·7, 13·1) μg/ml; *P* = 0·769).

Age (*P* < 0·001), sex (*P* = 0·011) and APACHE II score at admission (*P* < 0·001) were each significantly associated with resistin concentrations in the entire cohort. Mean resistin concentrations were lower in males than females, and males in the EN group had higher resistin concentrations than males in the NBM group. Conversely, females in the EN group had lower resistin concentrations than females in the NBM group. Further analysis showed, however, that after adjusting for age, sex and APACHE II score simultaneously, these confounders were not significantly associated with resistin concentrations between the two study groups (adjusted MD: 2·1 (95 % CI −1·4, 5·6) ng/ml; *P* = 0·241).

Sex was significantly associated with visfatin concentrations (*P* = 0·039) in the entire cohort. Males had a higher mean visfatin concentration than females in both groups. There was a significant interaction between APACHE II score at admission and visfatin concentration between the two study groups (*P* = 0·019). A positive correlation was observed between visfatin and APACHE II score at admission in the group who received EN, while an inverse correlation was observed between visfatin and APACHE II score at admission in the group who were on NBM. Further analysis demonstrated that after adjusting for sex, the MD in visfatin concentrations between groups was non-significant (adjusted MD: 0·2 (95 % CI −0·4, 0·8) ng/ml; *P* = 0·485).

## Discussion

This is the first study to examine adipokine concentrations in patients with AP who received EN compared with a NBM regimen. This is also the first study to test a comprehensive panel of five adipokines in AP. It demonstrates that EN significantly makes an impact on adipokine concentrations, specifically showing that commencement of EN within 24 h of hospital admission results in higher omentin concentrations and higher leptin concentrations compared with patients who were NBM. Early EN did not have a significant effect on adiponectin, resistin or visfatin concentrations. This study is important as it addresses potential mediators behind the beneficial effects of EN in AP. The identification of such mediators is needed in order to pave the way to use this information to best take advantage of the therapeutic effects of nutrition.

In addition, this is the first time omentin has been measured during AP in human subjects. Research in other disease states has provided insight that omentin, an adipokine secreted by adipose stromal vascular cells, exerts anti-inflammatory effects, with the findings that omentin concentrations are significantly decreased in obesity, insulin resistance, diabetes and CHD^(^^28^^–^^33^^)^.

A study in ninety-one healthy volunteers showed that those who were overweight or obese had significantly lower omentin concentrations than lean subjects, and that omentin concentrations were inversely correlated with BMI, waist circumference and insulin resistance^(^^30^^)^. A clinical study in forty-six patients with impaired glucose tolerance (IGT), fifty-five patients with newly diagnosed type 2 diabetes mellitus (T2DM), and fifty subjects with normal glucose tolerance (NGT) found that omentin concentrations were lower in patients with IGT and T2DM than patients with NGT, with the lowest concentrations observed in those with T2DM^(^^28^^)^. This finding is supported by two other clinical studies which found that omentin concentrations were significantly lower in patients with T2DM compared with healthy controls^(^^32^^,^^34^^)^. Furthermore, in a study of 201 men who underwent annual health check-ups, omentin concentrations were found to be inversely correlated with a higher number of metabolic risk factors including waist circumference, hypertension, dyslipidaemia and glucose intolerance^(^^29^^)^. Omentin levels also inversely correlate with nascent metabolic syndrome^(^^35^^,^^36^^)^. Thus, omentin may attenuate or prevent inflammatory responses in the metabolic syndrome and CVD.

In the setting of pancreatitis, omentin has only been studied in a rat model (eight controls, eight with AP, and eight with chronic pancreatitis)^(^^37^^)^. Results showed that omentin concentrations were significantly higher in rats with AP than control rats. The authors stated that elevated omentin concentrations cause a significant reduction in glucose levels, suggesting a potential role of omentin in glycaemic control^(^^37^^)^. Blood glucose control in patients with AP or other acute illnesses is affected by the route of feeding. A systematic review including 264 patients from six randomised controlled trials found that EN significantly reduces the risk of hyperglycaemia and the insulin requirement, thus providing better glucose control when compared with parenteral nutrition^(^^7^^)^. In the present study, levels of omentin were significantly higher in the group that received EN. It is therefore reasonable to speculate that the beneficial effect of early EN in patients with AP could be mediated, at least in part, by omentin and this could be achieved by modulating blood glucose homeostasis.

In the present study, patients who received EN also had significantly higher leptin levels, after adjusting for age, sex and BMI. Leptin, a hormone known to regulate food intake, metabolism and body weight, increases with food intake and decreases during fasting^(^^38^^,^^39^^)^. Thus, a raised leptin level in the EN group is an expected response to the stimulation of EN.

However, recent studies indicate that this relationship may be more complex than leptin being only a satiety factor; as it is now known to have effects on multiple organ systems^(^^40^^)^. A study in rats with cerulein-induced AP (conducted in parallel to fifteen humans with AP and thirty controls) showed that leptin concentrations were significantly higher in humans and rats with AP, compared with controls^(^^40^^)^. There was an up-regulation of leptin microRNA and protein in the rat pancreas after induction of AP, which could indicate that the pancreas is a source of local leptin production. Pre-treating with 10 µg/kg of exogenous leptin prior to the induction of AP attenuated dose-dependently the severity of the immune response in the pancreas, with reduced histological damage, reduced TNF-α, increased IL-4, and reduced expression of NO synthase^(^^40^^)^. Experimental studies in rats with AP have also found leptin receptors in the islet cells of the pancreas, suggesting that leptin may have a role in the control of glucose homeostasis^(^^41^^,^^42^^)^. These findings contrast with the pro-inflammatory effects of leptin in other tissues and may indicate a tissue-specific anti-inflammatory role of leptin in AP^(^^43^^)^. The leptin receptor has not yet been found in the human pancreas and identification of it would be important in determining the potential role of leptin in the inflammatory response in AP.

There are some limitations to this study. First, the relatively small sample size may prevent possible associations in this study from being uncovered. Yet, to date, this is the largest study to investigate a comprehensive panel of adipokines in patients with AP. Second, although significant differences in omentin and leptin concentrations were detected between the two study groups, these need to be interpreted with caution due to small discrepancies in the number of samples available for analysis on each day in each group for omentin and rather small absolute MD (2·3 ng/ml) in leptin concentration between the two groups. Last, the study population was limited to patients with mild to moderate AP at the time of enrolment, as defined by the determinant-based classification of AP severity^(^^44^^,^^45^^)^. Further studies are needed to confirm our findings in patients with severe and critical AP.

In conclusion, the present study identified that omentin and leptin concentrations increase in response to EN, which may mediate beneficial effects through anti-inflammatory mechanisms and may be protective against the development of insulin resistance due to the role of these adipokines in insulin-signalling pathways. These adipokines could also be important mediators involved in the modulation of inflammatory responses, and monitoring of their levels early in the course of AP may be justified due to their involvement in a complex interlinked network between nutrition, AP and blood glucose control. This research opens up a window into another chapter behind the mechanistic benefits of EN, to complement and challenge the notion that the benefits are solely due to improvement in gut barrier function.
